# Multiple mutations in the *EPSPS* and *ALS* genes of *Amaranthus hybridus* underlie resistance to glyphosate and ALS inhibitors

**DOI:** 10.1038/s41598-020-74430-0

**Published:** 2020-10-19

**Authors:** Maria J. García, Candelario Palma-Bautista, José G. Vazquez-Garcia, Antonia M. Rojano-Delgado, María D. Osuna, Joel Torra, Rafael De Prado

**Affiliations:** 1 Department of Botany, Ecology and Plant Physiology, University of Cordoba, 14071 Córdoba, Spain; 2 Department of Agricultural Chemistry and Edaphology, University of Cordoba, 14071 Cordoba, Spain; 3 Agrarian Research Center ‘Finca La Orden’ Valdesequera, Badajoz, Spain; 4 Departament D’Hortofructicultura, Botànica I Jardineria, Agrotecnio, Universitat de Lleida, 25198 Lleida, Spain

**Keywords:** Plant sciences, Plant breeding

## Abstract

*Amaranthus hybridus* is one of the main weed species in Córdoba, Argentina. Until recently, this weed was effectively controlled with recurrent use of glyphosate. However, a population exhibiting multiple resistance (MR2) to glyphosate and imazamox appeared in a glyphosate resistant (GR) soybean field, with levels of resistance up to 93 and 38-fold higher to glyphosate and imazamox, respectively compared to the susceptible (S) population. In addition to imidazolinones, MR2 plants showed high resistance levels to sulfonylamino-carbonyl (thio) benzoates and moderate resistance to sulfonylureas and triazolopyrimidines. Multiple amino acid substitutions were found in both target genes, acetolactate synthase (ALS) and 5-enolpyruvylshikimate-3-phosphate synthase (EPSPS), responsible for conferring high herbicides resistance levels in this *A. hybridus* population. In the case of *EPSPS*, the triple amino acid substitution TAP-IVS was found. In addition, MR2 plants also showed increased *EPSPS* gene expression compared to susceptible plants. A Ser653Asn substitution was found in the *ALS* sequence of MR2, explaining the pattern of cross-resistance to the ALS-inhibitor herbicide families found at the ALS enzyme activity level. No other mutations were found in other conserved domains of the *ALS* gene. This is the first report worldwide of the target site resistance mechanisms to glyphosate and ALS inhibitors in multiple herbicide resistance *Amaranthus hybridus*.

## Introduction

Triazine- and dinitroaniline-resistant weeds that evolved in the 1970s and 1980s were controlled by addition or replacement with acetolactate synthase (ALS) and acetyl-coenzyme A carboxylase (ACCase) inhibitors in the 1980s and 1990s. In fact, with the introduction of soybean in South America, farmers used IMI herbicides (imidazolinones) as their first chemical option in pre-planting and post-emergence of soybean. This high selection pressure led to the emergence of ALS-resistant populations in the late 1990s. Between 1993 and 2004, resistance to ALS was reported for *Bidens pilosa, Bidens subalternans*, *Euphorbia heterophylla* and *Amaranthus hybridus* in countries such as Brazil, Paraguay and Argentina^1,2^, and these biotypes are now widespread. ALS- and ACCase inhibitor-resistant weeds were controlled by the addition of protoporphyrinogen oxidase (PPO) inhibitors or glyphosate in glyphosate-resistant (GR) crops^3^.

Farmers in South America quickly adopted technology packages for GR (glyphosate resistant) crops, mainly soybeans and corn. Ninety percent of these two main crops in this region are transgenic GR crops^4,5^. The low costs of technology packages and the limited use of herbicides, specifically in plants affected by glyphosate, allowed them to be very competitive in the world market. The basis of this new tool was the use of residual herbicide imazethapyr (imidazolinone) and subsequently glyphosate in post-emergence of weeds. For a decade, glyphosate was efficiently used to control *Amaranthus* species, but since 2016, an *A. hybridus* population multiple resistant to synthetic auxins and glyphosate has appeared in GR soybean fields south of Cordoba, Argentina^8^. Abuse of herbicides in GR crops has led to the appearance of a wide range of superweeds resistant mainly to the herbicide glyphosate^5^.

Various attributes such as its high growth rate, high fertility, high genetic variability, stress tolerance, and the ability to evolve herbicide resistance confer to *Amaranthus* species the ability to become a dominant weeds that are difficult to control in summer crops^6,7^. Herbicide resistance in *Amaranthus* species becomes worrying due to the evolution of multiple resistant biotypes in recent years^2^. Among them *A. hybridus* is an annual broad-leaved species of American origin, today distributed throughout this continent^7^. There are 32 reports of resistance cases worldwide for this species to herbicides with different sites of action, such as Photosystem II (PSII), ALS, PPO and 5-enolpyruvylshikimate-3-phosphate synthase (EPSPS) inhibitors, as well as synthetic auxins^2^.

Target-site resistance-related *EPSPS* mechanisms have been described in different species from the *Amaranthus* genus. The first case of glyphosate resistance induced by *EPSPS* gene copy number occurred in an *A. palmeri* population in 2010^9^. A few years later, in 2014, this mechanism was also found in *A. spinosus*^10^ and *A. tuberculatus*^11,12^; in these cases, high *EPSPS* gene copy numbers were correlated with *EPSPS* overexpression. In addition to increased gene copy number and gene expression, another target-site resistance mechanism is mutation. Single and double mutations at Pro106^13^ and Thr102^14,15^ in the conserved domain of the *EPSPS* gene have also been described in several weed species. Finally, a triple amino acid substitution in the conserved region of the *EPSPS* gene (^95^LFLGNAGTAMRPL^107^), involving Thr102Ile, Ala103Val and Pro106Ser, has been very recently described as the sole mechanism responsible for a high level of resistance in two *A. hybridus* populations from Córdoba and Santa Fe, Argentina^16,17^.

Regarding ALS, the main molecular resistance mechanism described is the target-site mutation. To date, 28 amino acid substitutions endowing ALS resistance at 8 positions (Ala122, Pro197, Ala205, Asp376, Arg377, Trp574, Ser653, and Gly654) of the *ALS* gene in weed species have been identified^18,19^, with the Pro197 mutation being the most frequently observed.

In *Amaranthus hybridus,* a Trp574Leu substitution was found in 2004 in populations from Massac and Pope Counties, Illinois^20^. A few years later, a new mutation in the *ALS* gene, Ala122Thr, was also described in *A. hybridus* from Illinois^21^. In 2006, the Ser653Asn amino acid substitution was described in several populations from USA^22^. In 2007, a new amino acid substitution in the *ALS* gene at the Asp376 position was described in a resistant *A. hybridus* population from USA, conferring resistance to the five classes of ALS-inhibiting herbicides, namely, imidazolinones (IMIs), triazolopyrimidines (TPs), pyrimidinyl (thio) benzoates (PTBs), sulfonylamino-carbonyl triazolinones (STs) and sulfonylureas (SUs)^23^. Additionally, very recently, two independent mutations in the ALS gene sequence, namely, Trp574Leu and Asp376Glu, were found in two *A. hybridus* populations from Santa Fe and Cordoba, Argentina^24^ and in *A. palmeri* population from Brazil, in this case the substitution showed were Trp574Leu and Ser653Asp^25^. The Trp574Leu mutation is known to confer resistance to both imidazolinones (IMIs) and sulfonylureas (SUs), while the Ser653Asn mutation is known to confer resistance to only IMIs^26–30^.

Characterizing the mechanisms that confer single, multiple or cross resistance in a weed population is a very valuable information tool because it directly influences correct decision-making on weed management. Furthermore, the great diversity of resistance mechanisms described highlights the dangers of extrapolating the knowledge obtained from one resistant population to others. This work is focused on the molecular characterization of the target-site resistance (TSR) mechanisms in an *Amaranthus hybridus* population collected in a GR soybean field from Cordoba (Argentina). Putative resistance to glyphosate and different ALS inhibitors was studied to decipher its TSR mechanisms conferring multiple resistance to glyphosate, imazamox, tribenuron, florasulam, flucarbazone and byspiribac.

## Materials and methods

### Plant material

Mature seeds of an *A. hybridus* population suspected of having multiple resistance (MR) to imazamox and glyphosate were collected from 25 plants in a soybean field (RR) that had been treated with IMI (imidazolinone) herbicides and glyphosate for 20 years in an area of the campus of the University of Córdoba (Argentina). The seeds of a susceptible population (S) were also collected from 25 plants in 2016 from a nearby garden (300 m between the MR and S plants) in which no herbicide had ever been applied.

Multiple resistance in *A. hybridus* was corroborated for the MR population. For this purpose, seeds were germinated, and a thousand seedlings were transplanted in plots (2 m × 5 m) in the experimental field of the University of Córdoba (Spain). The herbicides were applied using a Pulverex backpack sprayer (Agrocor SA, Córdoba, Spain) with a T-coupler for the bar equipped with four flat fan nozzles, which were calibrated to deliver 200 L ha^-1^ at a spray pressure of 200 kPa at a height of 50 cm from the level of the plant. When the plants reached the four-leaf stage, they were treated with imazamox at 40 g ai ha^-1^. Two weeks later, the surviving plants were treated with glyphosate at 1080 g ae ha^-1^. Three months later, more than 80% of the plants finished their reproductive cycle, and mature seeds with multiple resistance were harvested and used for the next step. Approximately 250 seedlings were transplanted in trays (40 cm × 60 cm × 15 cm) that contained sand/peat in a ratio of 1:1 (v/v), and the growth and application followed the same conditions described above. Approximately 95% of the treated plants finished their reproductive cycle. Three months later, the mature seeds were collected, cleaned and stored at 4 °C for one month, and the new seeds were designated as the MR2 population. In parallel, 250 seedlings of the population susceptible to herbicides were treated with imazamox (20 g ai ha^-1^) and glyphosate (360 g ae ha^-1^) separately as explained above. Twenty-one days after the application of imazamox and glyphosate separately, 100% of the plants died, indicating the sensitivity to both herbicides. Both MR2 and S seeds were used in dose–response, biochemical and molecular studies to characterize the multiple resistance.

Seeds from the S and MR2 populations were germinated in Petri dishes containing filter paper that was moistened with distilled water. The Petri dishes were placed in a growth chamber at 28/18 °C (day/night) with a photoperiod of 16 h, 850 μmol s^-1^ photosynthetic photon flux and 80% relative humidity. All seedlings were transplanted in pots (one plant per pot) containing sand/peat at a ratio of 1:1 (v/v) and placed in a greenhouse with the same photoperiod.

### Dose–response assays

To quantify the level of glyphosate and imazamox resistance, seeds of the susceptible (S) and resistant (MR2) populations were germinated as described previously in the Plant material section. Plants from the S and MR2 *A. hybridus* populations were treated at the 4-leaf growth stage by using a laboratory system (SBS-060 De Vries Manufacturing, Hollandale, MN, USA) equipped with 8002 flat fan nozzles delivering 200 L ha^-1^ at a height of 50 cm from the plant level. Glyphosate (Roundup Energy® SL, 480 g ae L^-1^ as isopropyl amine salt, Monsanto) was applied at 8 doses (10 plants dose^-1^), namely, 0, 7.8, 15.6, 31.25, 62.5, 125, 250 and 500 g ae ha^-1^, to S plants and at 0, 31.25, 62.50, 125, 250, 500, 1000, 2000 and 4000 g ae ha^-1^ to MR2 plants. Another different group of S and MR2 plants was treated with imazamox (Pulsar® 40 SL, 4 g ai L^-1^) at different doses (10 plants dose^-1^): 0, 2.5, 5, 10, 20, 40 and 80 g ai ha^-1^ to S plants and 0, 40, 80, 160, 320, 640 and 1280 g ai ha^-1^ to MR2 plants. Nontreated plants were used as control. The experiments were organized in a completely randomized design and were repeated twice.

Plant mortality (LD_50_) and aerial part dry weight reduction (GR_50_) were determined at 28 days after treatment (DAT). Data are expressed as percentages in relation to the untreated controls.

### ALS and EPSPS enzyme activity assays

For both enzyme activity assays two steps were made: extraction and enzymatic reaction.

The ALS enzyme activity was measured as previously described Palma-Bautista et al.^31^ by mean the estimation of the product acetolactate after its conversion to acetoin, by decarboxylation in the presence of acid.

Seedling foliar tissue (3.0 g) from the 4–5 leaves growth stage, was frozen in liquid nitrogen and powdered with addition of polyvinylpolypyrrolidone (PVPP). The extraction buffer in proportion (4 mL g^-1^ tissue) was added. The extraction buffer contained 1 M potassium phosphate (KH_2_PO_4_ ⁄ K_2_HPO_4_), pH 7.5, 10 mM sodium pyruvate, 5 mM MgCl_2_, 50 mM thiamine pyrophosphate (TPP), 100 μM flavin adenine dinucleotide (FAD), 12 mM dithiothreitol (DTT) and glycerol (1:9, v ⁄v). This homogenate was shaken (10 min at 4ºC), filtered through four layers of cheesecloth, and centrifuged (15,000 g for 15 min at 4ºC). The supernatant obtained was immediately used for ALS enzyme activity assays.

ALS activity was assayed by adding 0.05 mL of enzyme extract to 0.1 mL of freshly prepared assay buffer [0.08 M potassium phosphate (KH_2_PO_4_ ⁄ K_2_HPO_4_), pH 7.5, 0.15 M sodium pyruvate, 1.5 mM MgCl_2_, 1000 μM FAD], and increased concentrations of technical-grade imazamox (0, 0.001, 0.01, 0.1, 10, 100, 1000, 10,000 µM). After mixture incubation (37 °C for 1 h), the reaction was stopped by addition of 0.05 mL of H_2_SO_4_ (3 M). The reaction tubes were then heated (15 min at 60 °C) to facilitate decarboxylation of acetolactate to acetoin. Acetoin was detected as a colored complex (520 nm) formed after the addition of 0.25 mL of creatine (5 g L^-1^, freshly prepared in water) and 0.25 mL of α-naphthol (50 g L^-1^, freshly prepared in 5 M NaOH) and incubated (60 °C for 15 min). Background was determined using control vials, in which the reaction was stopped before the incubation, and subtracted.

The herbicides concentrations necessaries to reduce the ALS activity by 50% (I_50_) were estimated as has been referred before. The tested herbicides were byspiribac, florasulam, flucarbazone and tribenuron-methyl using the same concentrations as the imazamox (except for florasulam in which the maximum was 1000 µM). To know the resistance pattern, it was required determine the relationship between the R I_50_ and the S I_50_ (R I_50_/S I_50_), as well as the biotypes resistance factor to each herbicide in-vitro assay. Total protein content was measured using the Bradford method^32,33^. Maximum ALS specific activity (nmol of acetoin mg^-1^ of protein h^-1^) was determined in the absence of herbicide and expressed as percentage in relation to the control.

For the EPSPS enzyme activity assay the methodology described by Salas et al.^34^ was followed. Five grams of leaf tissue from each population were finely powdered and transferred to tubes with 100 mL of cold extraction buffer (100 mM MOPS, 5 mM EDTA, 10% glycerol, 50 mM KCl and 0.5 mM benzamidine), 70 μL of β-mercaptoethanol and 1% PVPP. After agitation and subsequent centrifugation, (NH_4_)_2_SO_4_ in proportion 45% (w/v) was added to the supernatant, the mixture was stirred and then centrifuged. The previous step was repeated to precipitate the protein. All pellets were dissolved in 3 mL of extraction buffer and dialyzed in 2 L of dialysis buffer (30 mm, 1000-MWC dialysis tubing at 4ºC on a stir plate) over 12 h in cold chamber.

For the determination of EPSPS activity the EnzCheck phosphate assay Kit (Invitrogen, Carlsbad, CA). The substrates for the EPSPS enzyme reaction were phosphoenolpyruvate (1.02 mM) and shikimate-3-phosphate (0.41 mM), supplied by Sigma-Aldrich (Madrid, Spain). The assay buffer was composed of 1 mM MgCl_2_, 10% glycerol, 100 mM MOPS, 2 mM sodium molybdate and 200 mM NaF. The EPSPS activity from the populations was determined without and with glyphosate (0, 0.1, 1, 10, 100 and 1000 μM)32,33. EPSPS activity was measured for 10 min at 360 nm in a spectrophotometer (DU-640, Beckman Coulter Inc. Fullerton, USA) to determine the amount of inorganic phosphate (μmol) released, measured in μg^-1^ total soluble protein (TSP) min^-1^. The proteins concentration was determined by the Bradford assay^33^.

All experiments were conducted with three technical replication of each population per glyphosate and ALS-inhibiting herbicide concentration and repeated three times. EPSPS and ALS enzyme activity were expressed as percentage of enzyme activity in presence of glyphosate or ALS-inhibiting herbicide, respectively, with respect to the control.

### EPSPS and ALS copy number and gene expression

*EPSPS* and *ALS* gene copy numbers in the *A. hybridus* genomic DNA were determined as previously described by Gaines et al.^9^. The DNA was purified using the Qiagen DNeasy Plant Mini Kit (Qiagen, Valencia, CA) according to the manufacturer’s instructions. Once extracted, the DNA concentration was quantified using NanoDrop™ 1000 spectrophotometers (Thermo Scientific) to ensure that the concentration and purity were sufficient for further assays.

Young leaf tissue from ten individuals of each *A. hybridus* population was collected. Total RNA was extracted using TRI Reagent® solution (Molecular Research Center, Inc., Cincinnati, OH) according to the manufacturer’s instructions. RNA was treated using the RNase-free DNase Set (Qiagen, Valencia, CA). M-MLV reverse transcriptase (Promega, Madison, WI) was used to generate cDNA with 3 μg of total RNA as template and random hexamers as primers. The cycle conditions were as follows: 37 °C for 1 h, 42 °C for 30 min, 50 °C for 10 min and 15 °C for 10 min.

The *EPSPS* and *ALS* primer pairs used for copy number and gene expression assays were previously described by Gaines et al.^9^. Reactions were performed by using a qRT-PCR Bio-Rad CFX Connect thermal cycler and the following amplification profile: 50 °C for 2 min; 95 °C for 10 min; 40 cycles of 95 °C for 15 s and 60 °C for 1 min; and 95 °C for 15 s. PCRs were set up in 20 µl of SYBR Green PCR Master Mix (BIO-RAD). The *ALS* and Actin genes were used as reference genes to normalize the *EPSPS* and *ALS* qRT-PCR results, respectively. The relative expression levels were calculated from the threshold cycle (Ct) values and the primer efficiencies by the Pfaffl method^35^.

To determine *EPSPS* and *ALS* gene copy numbers, the *ALS* and actin genes were examined as reference genes by the Pfaffl method^35^. Triplicate technical replications were used to calculate the mean and standard error of the increase in *EPSPS*/*ALS* gene copy number relative to the ALS/Actin copy number.

Standard dilution curves were prepared for each primer pair to confirm the appropriate efficiency of amplification (E = 100 ± 10%).

### EPSPS and ALS gene sequencing

To achieve the *EPSPS* and ALS gene sequencing, ten plants from the susceptible (S) and the resistant (MR2) population were used.

A 196-bp DNA fragment from the conserved region of the *EPSPS* gene was amplified by RT-PCR as previously described by García et al.^16^. *EPSPS* gene sequencing was conducted by the Sanger method at the SCAI (“Central Service for Research Support”) of the University of Córdoba.

A pair of primers previously reported by Osuna et al.^36^ was used to amplify the *A. hybridus* ALS gene fragments containing all the known mutations. Sequencing of the purified genomic DNA was performed in the Genomic Unit Investigation Central Service of Badajoz University, Spain.

### Statistical analysis

The herbicide concentration that caused 50% dry weight reduction (GR_50_) and plant mortality (LD_50_) and the herbicide concentration that caused 50% inhibition of enzyme activity (I_50_) were calculated by analysis of nonlinear regression using the following logistic equation: *Y* = c + {(d-c)/[1 + (x/g)^b^]}, where *Y* is the dry weight, mortality and enzymatic inhibition in relation to the control, *d* is the coefficient corresponding to the limit of the upper asymptote, c is the limit of the coefficient of the lower asymptote (fixed at 0 for GR_50_ and LD_50_), *b* is the slope of the curve,* g* is the herbicide concentration required to inhibit shoot growth by 50%, and *x* is the herbicide dose^37^. Nonlinear regression analysis was performed in the R program 3.6.2 with the *drc* package (Statistical Software)^37,38^. The resistance indices (RI = MR2/S) were computed as MR2-to-S ratios.

## Results

### Dose–response assays

**Figure 1:**
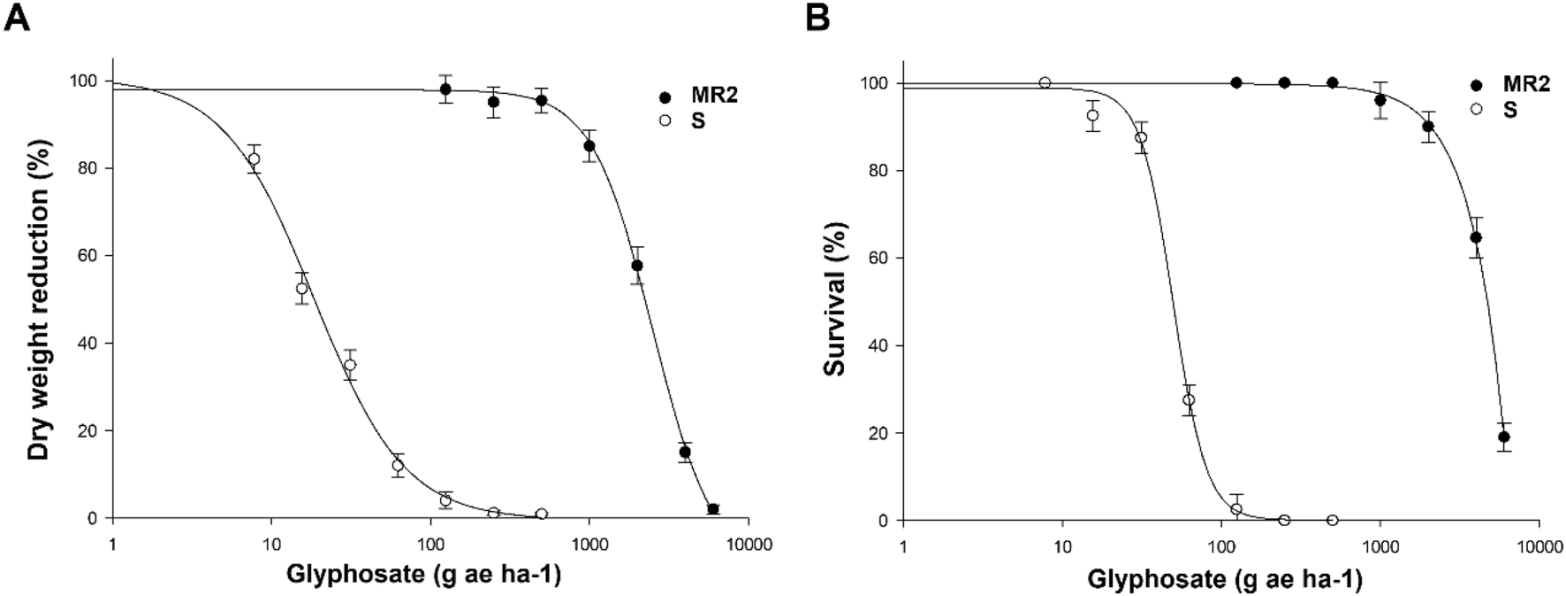
Glyphosate dose–response of dry weight (**A**) and survival (**B**) expressed as percentage of the mean dry weight (MR2 0.371 g^-1^ and S 0.369 g^-1^) of untreated control plants. The vertical bars represent the standard error of the mean (n = 10).

*Amaranthus hybridus* plants of the population MR2 exhibited resistance to glyphosate and imzamox in dose–response experiments. As shown in Fig. 1, plant survival and dry weight decreased as the dose of glyphosate increased. The susceptible population was eliminated by using lower glyphosate doses than those commonly used by Argentinian farmers (960 g ae ha^−1^). The GR_50_ and LD_50_ values estimated for the MR2 population were 2222 and 4508 g ae ha^−1^ glyphosate, respectively. According to the GR_50_ parameter, the RI of the MR2 population was 126 times higher than that of the S population (Table 1).

**Table 1:** Parameters of the log-logistic equations^a^ used to calculate the glyphosate rates required for 50% dry weight (GR_50_) and reduction survival (LD_50_), expressed as percentage of the mean untreated control of the *A. hybridus* population.

Growth reduction (GR_50_)					
Population	D	b	GR_50_ (g ae ha^-1^) ± SE	*P-*value	RI^b^
MR2	97.0	2.9	2222.0 ± 49.6	< 0.001	125.5
S	100.8	1.5	17.7 ± 0.8	< 0.001	–

Plant survival (LD_50_)					
Population	d	b	LD_50_ (g ae ha^-1^) ± SE	*P-*value	RI
MR2	98.8	4.7	4508.3 ± 57.2	< 0.001	93.6
S	100.9	4.1	48.2 ± 3.7	< 0.001	–

^a^ *Y* = c + {(d-c)/[1 + (x/g)^b^]}, where *d* is the coefficient corresponding to the upper asymptote, *c* is the limit of the coefficient of the lower asymptote (fixed at 0 for GR_50_ and LD_50_), *b* is the slope of the line, *x* is the herbicide dose, and g is the dose at the inflection point and hence the GR_50_ or LD_50_. ± SE is the standard error of the mean (n = 10)*.* The *P*-value is the level of significance of the non-linear regression model.

^b^RI (resistance index) = GR_50_, or LD_50_ (MR2)/GR_50_, or LD_50_ (S).

**Figure 2:**
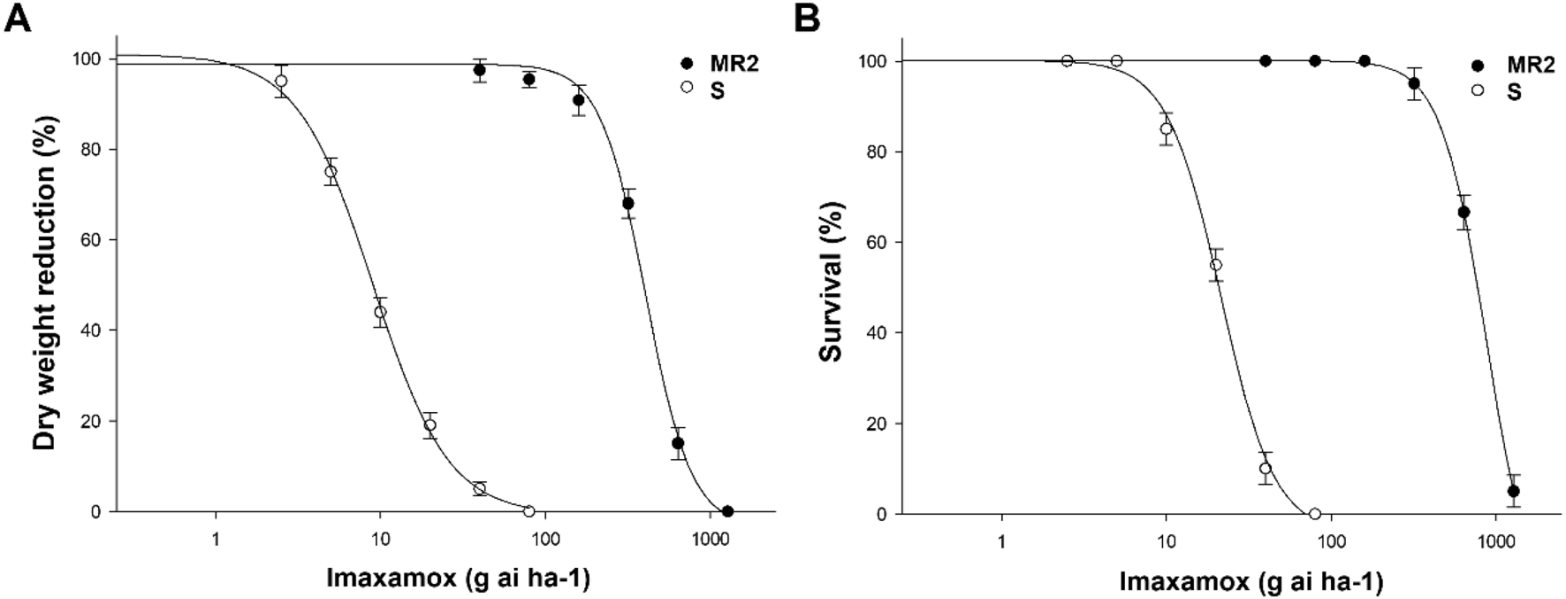
Imazamox dose–response of dry weight (**A**) and survival (**B**) expressed as percentage of the mean dry weight (MR2 0.372 g^-1^ and S 0.371 g^-1^) of untreated control plants. The vertical bars represent the standard error of the mean (n = 10).

For the imazamox herbicide (field dose 40 g ai ha^-1^), the results obtained were similar to those obtained with glyphosate; as the imazamox dose increased, plant survival and dry weight decreased (Fig. 2), and the GR_50_ and LD_50_ values obtained for the MR2 populations were 403 and 798, respectively. In this case, the RI of the MR2 population was 39, indicating that the MR2 population was 46-fold more resistant to imazamox than the S population (Table 2).

**Table 2:** Parameters of the log-logistic equations^a^ used to calculate the imazamox rates required for 50% dry weight (GR_50_) and reduction survival (LD_50_), expressed as percentage of the mean untreated control of the *A. hybridus* population.

Growth reduction (GR_50_)					
Population	d	b	GR_50_ (g ai ha^-1^) ± SE	*P-*value	RI^b^
MR2	95.8	3.4	403.5 ± 9.7	0.010	45.6
S	101.1	1.9	8.9 ± 0.2	< 0.001	–

Plant survival (LD_50_)					
Population	d	b	LD_50_ (g ai ha^-1^) ± SE	*P-*value	RI
MR2	100.2	3.1	798.4 ± 45.3	< 0.001	38.6
S	99.7	2.9	20.7 ± 1.4	< 0.001	–

^a^*Y* = c + {(d-c)/[1 + (x/g)^b^]}, where *d* is the coefficient corresponding to the upper asymptote, *c* is the limit of the coefficient of the lower asymptote (fixed at 0 for GR_50_ and LD_50_), *b* is the slope of the line, *x* is the herbicide dose, and *g* is the dose at inflection point and hence the GR_50_ or LD_50_.** ± **SE is the standard error of the mean (n = 10). The *P*-value is the level of significance of the non-linear regression model.

^b^RI (resistance index) = GR_50_ or LD_50_ (MR2)/GR_50_, or LD_50_ (S).

### EPSPS and ALS enzyme activity assays

**Figure 3:**
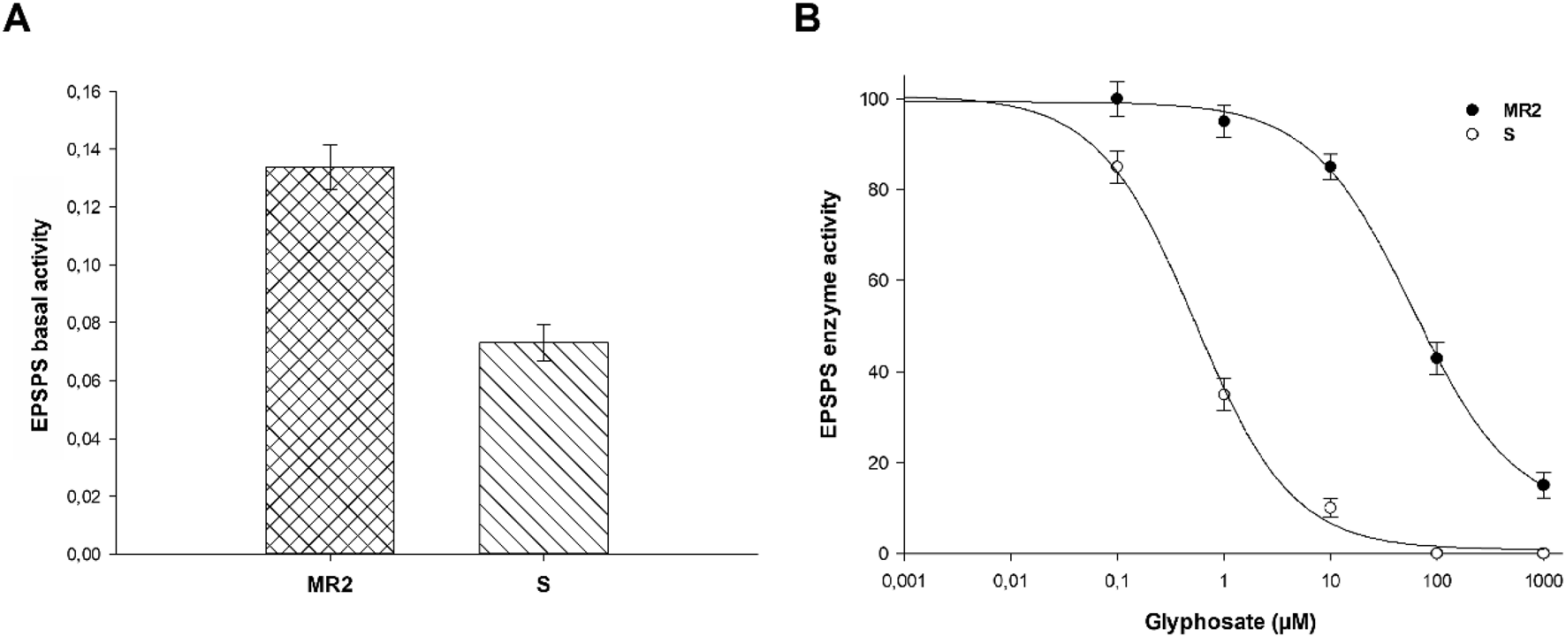
EPSPS activity in MR2 and S populations of *A. hybridus* from Córdoba, Argentina. (**A**) EPSPS enzyme basal activity, Histograms represent the means ± SEs (n = 9). (**B**) Dose–response curves of EPSPS enzyme activity expressed as a percentage of the untreated control. Mean ± SE. (n = 9).

The EPSPS basal activity profiles in the S and MR2 *A. hybridus* plants differed significantly (Fig. 3A). As shown in Fig. 3B, EPSPS activity was inhibited in both S and MR2 plants as glyphosate concentrations increased, but this EPSPS activity inhibition occurred at different herbicide concentrations (Fig. 3B), namely, 0.5 and 59 µM glyphosate for the S and MR2 populations, respectively, resulting in an RI (R-to-S ratio) of 111 (Table 3).

**Table 3:** Parameter estimates of the equation^a^ used to calculate the glyphosate concentration (µM) needed to reduce the activity of the EPSPS enzyme by 50% (I_50_) in the two *Amaranthus hybridus* biotypes (MR2 and S).

Species	Biotype	c	d	b	I_50_ (μM) ± SE	*P*-value	RI^b^
*A. hybridus*	MR2	8.5	99.3	0.9	59.1 ± 6.7	< 0.001	111.5
	S	0.8	100.6	1.0	0.5 ± 0.0	< 0.001	–

^a^*Y* = *c* + {(*d*–*c*)/[1 + (*x*/*g*)^*b*^]}, where *c* and *d* are the coefficients corresponding to the upper and lower asymptote, respectively; *x is* the glyphosate concentration; *b* is the slope of the line; and *g* is the glyphosate concentration at inflection point (I_50_). ± SE is the standard error of the mean. The *P*-value is the level of significance of the non-linear regression model.

^b^RI (resistance index) = I_50_ MR2/I_50_ S.

**Figure 4:**
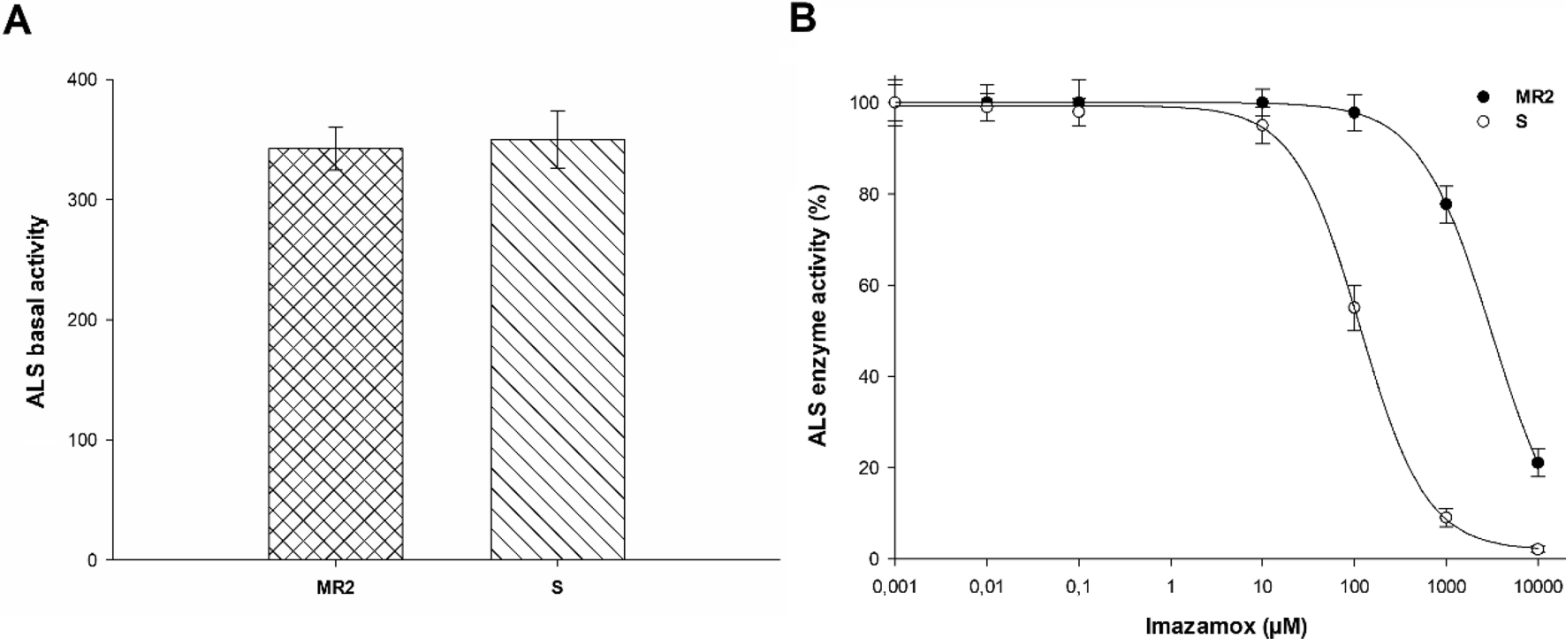
ALS activity in MR2 and S populations of *A. hybridus* from Córdoba, Argentina. (**A**) ALS enzymatic basal activity, Histograms represent the means ± SE (n = 9). (**B**) Dose–response curves of ALS enzymatic activity expressed as a percentage of the untreated control. Mean ± SE. (n = 9).

The *A. hybridus* S and MR2 plants presented similar ALS basal activity profiles (Fig. 4A). Imazamox inhibited ALS activity in both cases being the dose needed to do that higher in MR2 plants (Fig. 4B). These concentrations were 117 and 3171 µM imazamox for the S and MR2 populations, respectively, resulting in an RI (R-to-S ratio) of 27. (Table 4).

**Table 4:** Parameter estimations of the equation used to calculate the concentration (µM) of the ALS inhibitor herbicides necessary to reduce the activity of the ALS enzyme by 50% (I50) in the two *Amaranthus hybridus* biotypes (MR2 and S).

Herbicide	Population	c	d	b	I_50_ (μM) ± SE	*P*-value	RI^b^
Tribenuron	MR2	1.5	97.7	0.7	272.7 ± 26.0	< 0.001	5.7
	S	0.1	94.2	0.8	47.5 ± 6.3	0.008	–
Florasulam	MR2	− 0.2	99.1	0.8	56.5 ± 9.0	< 0.001	3.2
	S	1.9	99.0	1.5	17.4 ± 1.4	< 0.001	–
Flucarbazone	MR2	0.7	98.6	1.2	144.7 ± 11.5	< 0.001	149.2
	S	0.9	98.3	0.8	1.0 ± 0.1	< 0.001	–
Byspiribac	MR2	1.7	98.4	0.6	2728.4 ± 80	0.011	59.5
	S	− 0.9	98.7	0.8	45.9 ± 8.0	< 0.001	–
Imazamox	MR2	− 1.5	100.0	1.1	3171.8 ± 111.9	0.016	27.1
	S	1.9	99.3	1.2	117.0 ± 9.1	< 0.001	–

^a^ *Y* = *c* + {(*d*–*c*)/[1 + (*x*/*g*)^*b*^]}, where *c* and *d* are the coefficients corresponding to the upper and lower asymptote, respectively; x herbicide concentration; *b* is the slope of the line; and *g* is the herbicide concentration at inflection point (I_50_). ± SE is the standard error of the mean. The *P*-value is the level of significance of the non-linear regression model.

^b^ RI (resistance index) = I_50_ MR2/I_50_ S.

### EPSPS and ALS copy number and gene expression

**Figure 5:**
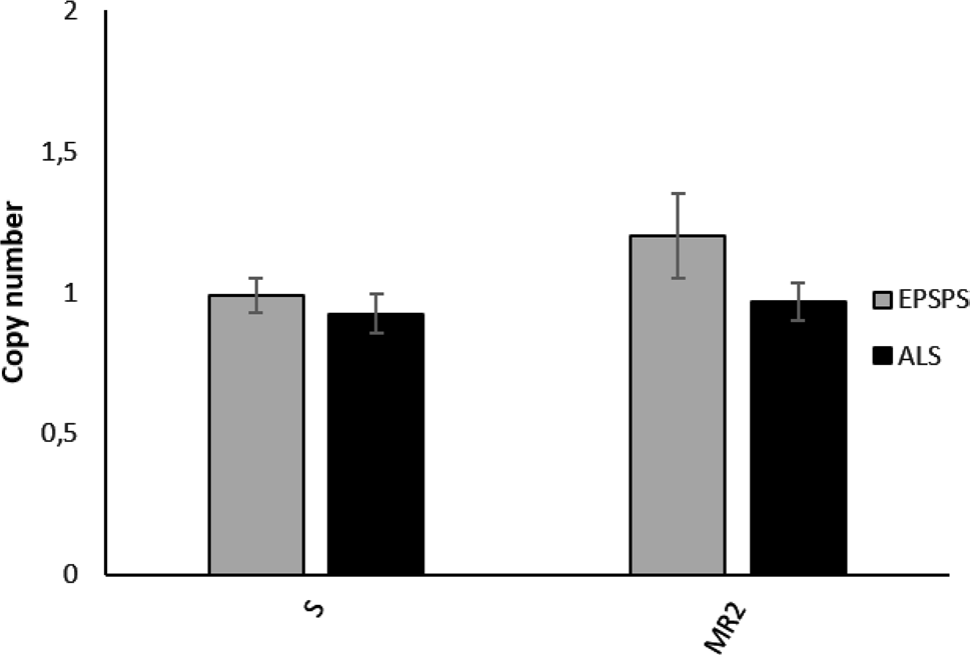
*EPSPS* and *ALS* gene copy numbers in *A. hybridus* S and MR2 populations from Córdoba, Argentina. Values represent the average of ten independent plants and the standard error.

**Figure 6:**
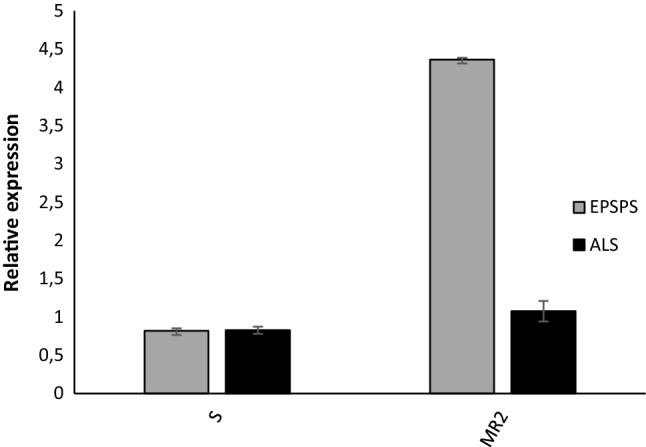
*EPSPS* and *ALS* relative gene expression in *A. hybridus* S and MR2 populations from Córdoba, Argentina. Values represent the average of ten independent plants and the standard error.

No significant differences were observed in the *EPSPS* and *ALS* gene copy numbers between S and MR2 *A. hybridus* plants (Fig. 5). Nevertheless, *EPSPS* gene expression was higher in MR2 plants compared to S population, with expression levels up to 4 times higher (Fig. 6). In most cases an increase in the expression levels of a gene is usually correlated with a higher copy number of the gene, but this is not the current case. In MR2 population the high *EPSPS* expression levels are not correlated with an *EPSPS* copy number increment, this suggests that *EPSPS* gene expression is subjected to a regulation mechanism at transcriptional level.

### Mutations in the EPSPS and ALS coding sequences

The point mutations found both in *EPSPS* and *ALS* genes corroborated the resistance to glyphosate and imazamox from whole plant experiments. The multiple resistant *A. hybridus* population showed the EPSPS triple amino acid substitution Thr102Ile, Ala103Val and Pro106Ser which was previously described by our group^16^ with another *A. hybridus* population that was resistant only to glyphosate, data not shown (GenBank accession number MG595171).

**Figure 7:**
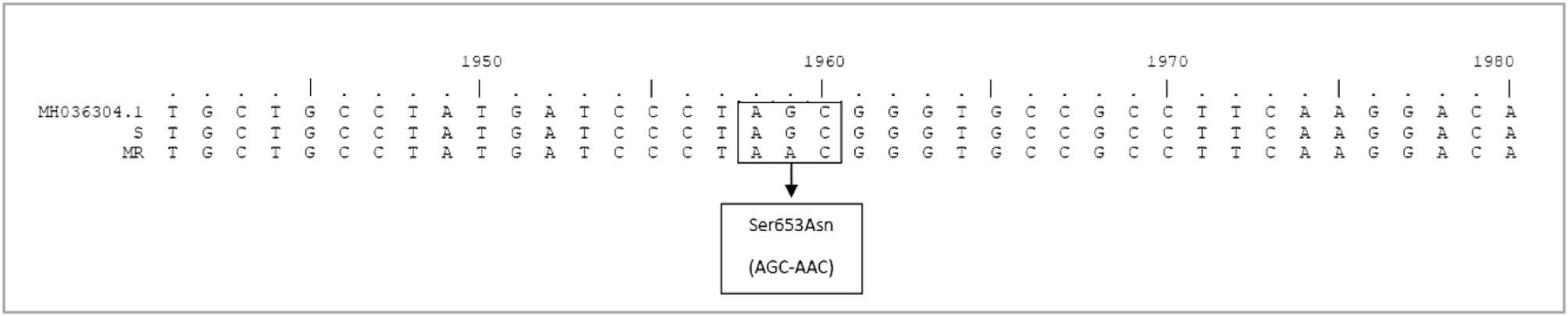
Sequence alignment of a region of the ALS gene from susceptible (S and GenBank: MH036304.1) and ALS-resistant (MR) *A. hybridus* populations. The sequences presented here show the site of domain E of the ALS gene. The amino acid numbering is based on the *Arabidopsis thaliana* ALS sequence.

Only a nucleotide point mutation (AGC to AAC) was found in the ALS sequence of MR2 when compared with the corresponding sequence in the S population, resulting in a Ser653Asn substitution (Fig. 7). This explains the pattern of cross-resistance to the ALS-inhibitor herbicide families found at the ALS enzyme activity level among these accessions. No other mutations were found in other conserved domains of the ALS gene (data not shown).

## Discussion

Multiple resistance to glyphosate and ALS inhibitor, namely imazethapyr, was previously found in an *A. hybridus* population from Argentina in 2014^2^. Several years later, in 2018, an *A. hybridus* population with multiple resistance to glyphosate and to the ALS inhibitor chlorimuron-ethyl, was described in Brazil^2^. Nevertheless, the resistance mechanisms were not described. In this study, multiple resistance to glyphosate and ALS inhibitors in the *A. hybridus* population from Córdoba, Argentina, has been studied and characterized to decipher the TSR mechanism underlying the high resistance levels shown for the first time. The GR_50_ value relative to glyphosate shown by this MR2 population (2222 g ae ha^−1^ glyphosate) was higher than that of the previous resistant population (Glyphosate-resistant) (1395 g ae ha^−1^ glyphosate), the results for which were previously published^16^. These two different populations, MR2 and GR2 (glyphosate resistant population which results were previously published in García et al.^16^) are carriers of the novel triple amino acid substitution in the conserved region of the *EPSPS* gene. The difference between them lies in the increased *EPSPS* gene expression shown by the MR2 population, which was up to 4 times higher in the MR2 population than in the S population. This increment in the *EPSPS* gene expression was not correlated with an increase in the *EPSPS* gene copy number. Assuming no differences between S and R plants in copy number, changes in transcriptional regulation might explain the *EPSPS* overexpression found in this particular population, which would be quite novel^16^. The first case of herbicide resistance induced by *EPSPS* gene copy number occurred in an *A. palmeri* population in 2010^7^. In this work, the authors found a 100-fold increase in *EPSPS* gene copy number, resulting in an up to 40-fold increase in *EPSPS* expression. These mechanisms, that is, *EPSPS* overexpression due to increased copy number, have also been described in other species of the *Amaranthus* genus, such as *A. tuberculatus*^12^ and *A. spinosus*^10^, as well as in multiple weed species^39,40^.

Mutations in the conserved region of *EPSPS* conferring glyphosate resistance have been previously reported in both mono- and dicotyledonous weeds. The single amino acid substitutions at Pro106 confer glyphosate resistance in several weeds, such as *Digitaria insularis, Lolium multiflorum, Amarantus spinosus, A. palmeri* and *Lolium rigidum*^9,41–43^. Single substitutions at Gly101 and Thr102 confer high levels of glyphosate resistance but decrease the volume of the glyphosate–PEP binding site, reducing affinity for PEP^44,45^. The artificial double amino acid change in *EPSPS* (Thr102Ile and Pro106Ser) known for conferring high glyphosate resistance levels in commercial transgenic maize lines, has also been reported in *Eleusine indica*^15,46^ and *Bidens pilosa*^47^. Very recently, Takano et al. (2020) found a double different substitution in EPSPS gene (Thr102Ile and Pro106Thr) in *Bidens subalternans* population from Paraguay^48^. The triple amino acid substitution in the *EPSPS* gene was reported for the first time in an *A. hybridus* population from Cordoba, Argentina^16^, and this mechanism was the only mechanism responsible for the high resistance level (GR_50_ of 1395 g ae ha^−1^) shown in this case^16^. The higher resistance level developed by the MR2 population (GR_50_ of 2222 g ae ha^−1^) must be due to the presence of an alternative second mechanism, as the *EPSPS* gene expression level was up to four times higher in MR2 plants than in S plants.

ALS point mutations have been widely described in numerous weed species, particularly at 8 positions of the ALS gene^19^. Depending on the ALS amino acid position affected and the specific substitution, variable patterns of cross-resistance between ALS inhibitor classes can occur^49^. Amino acid substitutions of Ala122 or Ser653 conferred resistance to IMI herbicides with low-level resistance to SUs^27,50–52^, whereas substitution of Pro197 conferred resistance to SUs^53^ but low or no cross-resistance to IMIs. Substitution of Trp574 or Ala205 conferred broad cross-resistance;^50,54^ however, substitution of Ala205 conferred much lower levels of resistance than substitution of Trp574.

In MR2 plants, the single amino acid substitution Ser653Asn is responsible for the high resistance level to IMIs. Although the Ser653 substitution has been described to confer resistance only to IMIs, there are some cases in which broad cross-resistance has been observed. Whaley et al.^22,23^ found that the Ser653Asn amino acid substitution conferred resistance to IMIs, pyrimidinyl (thio) benzoates (PTBs) and triazolopyrimidines (TPs) in several *Amaranthus hybridus* biotypes.

This is the first time that TSR mechanisms by means of point mutations for both glyphosate and ALS inhibitors are reported in *Amaranthus hybridus.* Moreover, an additional TSR mechanism to glyphosate has been found, because MR2 population showed increased *EPSPS* gene expression. In this regard, new management alternatives must be used, both chemical and non-chemical, to control this *A. hybridus* population from Córdoba, Argentina. Among chemical options, photosystem I and II, protoporphyrinogen oxidase (PPO) and 4-hydroxyphenylpyruvate dioxygenase (HPPD) inhibitors or auxin-mimic herbicides should be considered to ensure the sustainability of the crop production system.

## References

[1] Rojano-Delgado AM (2019). Target site as the main mechanism of resistance to imazamox in a 2 Euphorbia heterophylla biotype. Sci. Rep..

[2] Heap, I. The International Survey of Herbicide Resistant Weeds. Annual Report Internet. Available at, www.weedscience.org Accessed: March 2020. (2020).

[3] Heap I, Duke SO (2018). Overview of glyphosate-resistant weeds worldwide. Pest Manag. Sci..

[4] Peterson MA, Collavo A, Ovejero R, Shivrain V, Walsh MJ (2018). The challenge of herbicide resistance around the world: a current summary. Pest. Manag. Sci..

[5] Duke SO, Powles SB, Sammons RD (2018). Glyphosate - How it became a once in a hundred year herbicide and its future. Outlooks Pest Manag..

[6] Vitta JI, Faccini DE, Nisensohn LA (2000). Control of *Amaranthus quitensis* in soybean crops in Argentina: an alternative to reduce herbicide use. Crop Prot..

[7] Faccini D, Vitta JI (2005). Germination characteristics of *Amaranthus quitensis* as affected by seed production date and duration of burial. Weed Res..

[8] Dellaferrera I (2018). First report of *Amaranthus hybridus* with multiple resistance to 2,4-D, dicamba, and glyphosate. Agronomy.

[9] Gaines TA (2010). Gene amplification confers glyphosate resistance in *Amaranthus palmeri*. Proc. Natl. Acad. Sci. USA.

[10] Nandula VK (2014). *EPSS* amplification in glyphosate resistant spiny amaranth (*Amaranthus spinosus*): a case of gene transfer via interspecific hybridization from glyphosate-resistant Palmer amaranth (*Amaranthus Palmeri*). Pest Manag. Sci..

[11] Nandula VK, Ray JD, Ribeiro DN, Pan Z, Reddy KN (2013). Glyphosate Resistance in tall waterhemp (*Amaranthus tuberculatus*) from Mississippi is due to both Altered Target-Site and Nontarget-Site Mechanisms. Weed Sci..

[12] Lorentz L (2014). Characterization of glyphosate resistance in *Amaranthus tuberculatus* populations. J. Agric. Food Chem..

[13] Sammons RD, Gaines TA (2014). Glyphosate resistance: state of knowledge. Pest. Manag. Sci..

[14] Li J (2018). Glyphosate resistance in *Tridax procumbens* via a novel *EPSPS* Thr-102-Ser substitution. J. Agric. Food Chem..

[15] Yu Q (2015). Evolution of a double amino acid substitution in the 5-enolpyruvylshikimate-3-phosphate synthase in *Eleusine indica* conferring high-level glyphosate resistance. Plant Physiol..

[16] García MJ (2019). The triple amino acid substitution TAP-IVS in the *EPSPS* gene confers high glyphosate resistance to the superweed *Amaranthus hybridus*. Int. J. Mol. Sci..

[17] Perotti (2019). A novel triple amino acid substitution in the EPSPS found in a high-level glyphosate resistant *Amaranthus hybridus* population from Argentina. Pest Manag. Sci..

[18] Beckie HJ, Tardif FJ (2012). Herbicide cross resistance in weeds. Crop Prot..

[19] Tranel, P.J., Wright, T. R., Heap, I. M. Mutations in herbicide-resistant weeds to ALS inhibitors. Publishing PhysicsWeb: https://www.weedscience.com. Accessed 28 August 2019 (2019).

[20] Maertens KD, Sprague CL, Tranel PJ, Hines RA (2004). *Amaranthus hybridus* populations resistant to triazine and acetolactate synthase-inhibiting herbicides. Weed Res..

[21] Trucco F, Hager AG, Tranel PJ (2006). Acetolactate synthase mutation conferring imidazolinone-specific herbicide resistance in *Amaranthus hybridus*. J. Plant Physiol..

[22] Whaley CM, Wilson HP, Westwood JH (2006). ALS resistance in several smooth pigweed (*Amaranthus hybridus*) biotypes. Weed Sci..

[23] Whaley CM, Wilson HP, Westwood JH (2007). A new mutation in plant ALS confers resistance to five classes of ALS-inhibiting herbicides. Weed Sci..

[24] Larran AS, Lorenzetti F, Tuesca D, Perotti VE, Permingeat HR (2018). Molecular mechanisms endowing cross-resistance to ALS-inhibiting herbicides in *Amaranthus hybridus* from Argentina. Plant Mol. Biol. Rep..

[25] Küpper A (2017). Multiple resistance to glyphosate and acetolactate synthase inhibitors in Palmer Amaranth (*Amaranthus palmeri*) identified in Brazil. Weed Sci..

[26] Burgos NR, Kuk YI, Talbert RE (2001). *Amaranthus palmeri* resistance and differential tolerance of *Amaranthus palmeri* and *Amaranthus hybridus* to ALS-inhibitor herbicides. Pest Manag. Sci..

[27] Franssen AS, Skinner DZ, Al-Khatib K, Horak MJ, Kulakow PA (2001). Interspecific hybridization and gene flow of ALS resistance in Amaranthus species. Weed Sci..

[28] McCourt JA, Pang SS, King-Scott J, Guddat LW, Duggleby RG (2006). Herbicide-binding sites revealed in the structure of plant acetohydroxyacid synthase. Proc. Natl. Acad. Sci. USA.

[29] Powles SB, Yu Q (2010). Evolution in action: Plants resistant to herbicides. Annu. Rev. Plant. Biol..

[30] Tranel PJ, Wright TR (2002). Resistance of weeds to ALS inhibiting herbicides: what have we learned?. Weed Sci..

[31] Palma-Bautista C (2018). First case of *Conyza canadensis* from Hungary with multiple resistance to glyphosate and Flazasulfuron. Agronomy.

[32] Dayan FE (2015). Biochemical markers and enzyme assays for herbicide mode of action and resistance studies. Weed Sci..

[33] Bradford MM (1976). A rapid and sensitive method for the quantitation of microgram quantities of protein utilizing the principle of protein-dye binding. Anal. Biochem..

[34] Salas R.A., et al. EPSPS gene amplification in glyphosate resistant Italian ryegrass (*Lolium perenne* ssp. *multiflorum*) from Arkansas. *Pest Manag. Sci.* **68,**1223–1230 (2012). 10.1002/ps.3342 22815255

[35] Pfaffl MW (2001). A new mathematical model for relative quantification in real-time RT–PCR. Nucleic Acids Res..

[36] Osuna, M. D., Casado, C., Wargner, J., Hurle, K. and De Prado, R. A new mutation site in the acetolactate syntase (ALS) gene in *Amaranthus quitensis* resistant to imazethapyr. in *The BCPC International Congress*. 819–823 (2003).

[37] Ritz C, Baty F, Streibig JC, Gerhard D (2015). Dose-response analysis using R. PLoS ONE.

[38] R Core Team: A language and environment for statistical computing. R Foundation for Statistical Computing, Vienna, Austria. Available at, https://www.R-project.org/ (2020).

[39] Chahal PS, Varanasi VK, Jugulam M, Jhala AJ (2017). Glyphosate-resistant Palmer Amaranth (*Amaranthus palmeri*) in Nebraska: confirmation, *EPSPS* gene amplification, and response to POST corn and soybean herbicides. Weed Technol..

[40] Wiersma AT (2015). Gene amplification of 5-enol-pyruvylshikimate-3-phosphate synthase in glyphosate-resistant *Kochia scoparia*. Planta.

[41] De Carvalho LB (2012). Pool of resistance mechanisms to glyphosate in *Digitaria insularis*. J. Agric. Food. Chem..

[42] Gonzalez-Torralva F, Gil-Humanes J, Barro F, Brants I, De Prado R (2012). Target site mutation and reduced translocation are present in a glyphosate-resistant Lolium multiflorum Lam. biotype from Spain. Plant Physiol. Biochem..

[43] Fernandez P, Gauvrit C, Barro F, Menendez J, De Prado R (2015). First case of glyphosate resistance in France. Agron. Sustain. Dev..

[44] Funke T (2009). Structural basis of glyphosate resistance resulting from the double mutation Thr 97 → Ile and Pro 101 → Ser in 5-enolpyruvylshikimate-3-phosphate synthase from *Escherichia coli*. J. Biol. Chem..

[45] Eschenburg S, Healy M, Priestman M, Lushington G, Schönbrunn E (2002). How the mutation glycine96 to alanine confers glyphosate insensitivity to 5-enolpyruvyl shikimate-3-phosphate synthase from *Escherichia coli*. Planta.

[46] Chen J (2015). Mutations and amplification of *EPSPS* gene confer resistance to glyphosate in goosegrass (*Eleusine indica*). Planta.

[47] Alcántara-de la Cruz R (2016). Target and non-target site mechanisms developed by glyphosate-resistant Hairy beggarticks (Bidens pilosa L.) populations from Mexico. Front. Plant Sci..

[48] Takano, H. K. et al. A novel TIPT double mutation in *EPSPS *conferring glyphosate resistance in tetraploid *Bidens subalternans. Pest. Manag. Sci.* **76,** 95 13;102 (2020) 10.1002/ps.5535 31251461

[49] Shaner DL (1999). Resistance to acetolactate synthase (ALS) inhibitors in the United States: history, occurrence, detection and management. J. Weed Sci. Tech..

[50] Bernasconi P, Woodworth AR, Rosen BA, Subramanian MV, Siehl DL (1995). A naturally occurring point mutation confers broad range tolerance to herbicides that target acetolactate synthase. J. Biol. Chem..

[51] Devine, M. D. and Eberlein, C. V. Physiological, biochemical and molecular aspects of herbicide resistance based on altered target sites. Pages 159–185 in R. Michael Roe, J. D. Burton, and R. J. Kuhr, eds. Herbicide Activity: Toxicology, Biochemistry and Molecular Biology. Amsterdam: IOS (1997).

[52] Tranel, P.J., Wright, T.R, and Heap, I.M. Mutations in herbicide-resistant weeds to ALS inhibitors. Online https://www.weedscience.com. Accessed June 2020 (2020).

[53] Guttieri MJ, Eberlein CV, Mallory-Smith CA, Thill DC, Hoffman DL (1992). DNA sequence variation in domain A of the acetolactate synthase gene of herbicide resistant and susceptible weed biotypes. Weed Sci..

[54] Woodworth, A. R., Rosen, B. A., Bernasconi, P. Broad range resistance to herbicides targeting acetolactate synthase (ALS) in a field isolate of *Amaranthus sp*. is conferred by a Trp to Leu mutation in the ALS gene (Accession No. U55852) (PGR96–051). *Plant Physiol.* **111,**1353 (1996).

